# Machine Learning Techniques for Single Nucleotide Polymorphism—Disease Classification Models in Schizophrenia

**DOI:** 10.3390/molecules15074875

**Published:** 2010-07-12

**Authors:** Vanessa Aguiar-Pulido, José A. Seoane, Juan R. Rabuñal, Julián Dorado, Alejandro Pazos, Cristian R. Munteanu

**Affiliations:** Department of Information and Communication Technologies, Computer Science Faculty, University of A Coruña, Campus de Elviña, S/N, 15071 A Coruña, Spain; E-Mails: vanesa.aguiar@udc.es (V.A.-P.); jseoane@udc.es (J.A.S.); juanra@udc.es (J.R.R.); julian@udc.es (J.D.); apazos@udc.es (A.P.)

**Keywords:** DNA molecule, SNP, schizophrenia, artificial neural networks, evolutionary computation

## Abstract

Single nucleotide polymorphisms (SNPs) can be used as inputs in disease computational studies such as pattern searching and classification models. Schizophrenia is an example of a complex disease with an important social impact. The multiple causes of this disease create the need of new genetic or proteomic patterns that can diagnose patients using biological information. This work presents a computational study of disease machine learning classification models using only single nucleotide polymorphisms at the HTR2A and DRD3 genes from Galician (Northwest Spain) schizophrenic patients. These classification models establish for the first time, to the best knowledge of the authors, a relationship between the sequence of the nucleic acid molecule and schizophrenia (Quantitative Genotype – Disease Relationships) that can automatically recognize schizophrenia DNA sequences and correctly classify between 78.3–93.8% of schizophrenia subjects when using datasets which include simulated negative subjects and a linear artificial neural network.

## 1. Introduction

Disease computational studies use diverse types of data, such as the structure and physical/chemical properties of a protein and DNA/RNA molecules, blood proteome mass spectra, DNA microarray results, disease biomarkers and concentration of the metabolites in physiological liquids. Schizophrenia, which is a common disease, can be defined as a heterogeneous syndrome characterized by perturbations in language, perception, thinking, social relationships and will. There is not a set of symptoms which uniquely characterize the disease, and even though researchers have been looking for a unique cause of schizophrenia for years with no success, most of them have concluded that schizophrenia would be the consequence of several cumulative effects of certain risk factors (genetic and environmental) [1]. Several studies of families, twins and foster-children confirmed and have allowed quantification of the contribution of genetics to schizophrenia [2]. After this, molecular genetics techniques started to be used to identify the genes that caused the disease [3]. These genes are not the genes of schizophrenia themselves, but rather they may transmit a set of characteristics which would increase the risk of developing the disease.

One of the most studied genes in relation to schizophrenia susceptibility is DRD3. As well as HTR2A, it is considered to be an important target for several antipsychotic drugs [4,5]. HTR2A encodes one of the receptors for serotonin and DRD3 encodes one subtype of the five dopamine receptors, both neurotransmitters. More specifically, Dopamine 3 receptors (DRD3) are concentrated in limbic regions of the brain, which are associated with cognitive, emotional and endocrine functions. Thus, it may be particularly relevant to schizophrenia [6], as the DRD3 messenger RNA is predominantly expressed in the limbic system, a region thought to be dysfunctional in this disease [7,8].

Association studies involving these functional candidate genes have systematically focused on a limited set of Single Nucleotide Polymorphisms (SNPs), generally based on previously reported small contributions of these markers of risk of susceptibility to schizophrenia. More specifically, SNP T102C (rs6313) at HTR2A and SNP Ser9Gly (rs6280) at DRD3 have been extensively analyzed in several schizophrenia case-control studies [9]. A SNP [10] is a single nucleotide site where two (of four) different nucleotides occur in a high percentage (*i.e.,* at least 1 %) of the population.

There are several studies on SNPs, such as that one in [11], where a method is presented for haplotype partitioning based on pairwise analysis of SNPs. A block-based approach for mapping a single locus trait was applied to blocks of different methods in a case-control study. Results show that any block-based association test is considerably more efficient than the conventional single site association trait and, in particular, the method presented performed best accuracy, even when a low marker density was available. Another study on SNPs is that one presented in [12]. In this paper, the use of two feature importance ranking measures (the modified t-test and F-statistics) is proposed to rank a large amount of SNPs and then the greedy manner together with a classifier are used in order to determine a desirable feature subset, which leads to the highest classification accuracy with the minimum size. Results show that both ranking methods are efficient at determining the important SNPs and they both find nearly the same amount of them. However, the first measure tends to be better in terms of classification accuracy. Compared to other methods, the results obtained in this paper are better.

There exist several genetic data simulation packages. Among those, we encounter coalescent-based methods [13], which have been used for population based simulation in genetic studies, such as GENOME [14]. This method was developed to overcome previous limitations. HAP-SAMPLE [15], which is the simulator used in this paper, uses the existing Phase I/II HapMap data to resample existing phased chromosomes to simulate datasets. There also exist forward-time population simulations, such as easyPOP [16], FPG [17], FREGENE [18], simuPOP [19] and genomeSIMLA [20]. The last method can simulate realistic patterns of LD in both family-based and case-control datasets and, unlike other similar packages, has proved to be an effective platform for simulating large scale genetic data. Another program capable of generating large scale genetic and also phenotypic variation data is presented in [21]. This program generates genotypes/phenotypes by perturbing real data, with the aim of creating a large number of replicates that share similar properties with real data.

Models based on Machine Learning have been extensively used to analyze complex diseases, such as diabetes [22], hepatitis [23], rheumatoid arthritis [24], *etc*. However, not many studies have been carried out on variation analysis in schizophrenia using Machine Learning algorithms [25]. Statistical models were the most used for this type of complex disease.

**Figure 1:**
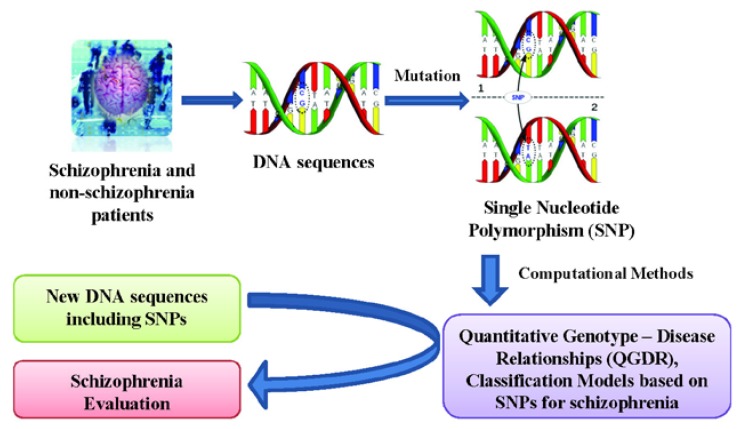
Flow chart of the QGDR model classification between the DNA structure (SNPs) and schizophrenia.

Quantitative Structure - Activity Relationships (QSARs) are widely used for predicting protein properties [26] and Quantitative Protein (or Proteome)-Disease Relationships (QPDRs) [27,28,29,30,31,32,33] for disease prediction. Recent works using complex networks of proteins or mass spectra of the human serum proteome have contributed to create theoretical models for cancer diagnosis and screening for cancer-related molecules in the case of colorectal [34,35], breast [34,36] and prostate [37,38,39] cancers. In a similar way, a Quantitative Genotype - Disease Relationship (QGDR) can be established in order to automatically evaluate schizophrenia DNA sequences using SNP data. Methods such as artificial neural networks [40], support vector machines [41], evolutionary computation [42,43] and other Machine Learning techniques [44] have been used in order to find the best classification models. This work presents a study of schizophrenia QGDR classification using only single nucleotide polymorphisms from Galician patients [9]. Thus, this information of the DNA molecule will be used as the input for several machine learning techniques that search for the best classification model capable of evaluating new schizophrenia DNA sequences (see Figure 1).

## 2. Results and Discussion

Two hundred and fifty two (252) QGDR classification models have been obtained using SNPs at two schizophrenia-related genes (each of them or both), twelve machine learning techniques and seven datasets, starting from the original data and using extra simulated negative (control) subjects (see Table 1). In terms of classification the subjects are organized in two groups: *Schizo* and *non-Schizo*. These models describe relationships between the DNA information (SNPs) and schizophrenia. 

**Table 1:** The classification models obtained for the evaluated schizophrenia patients using the SNP information at DRD3 and HTR2A.

Data set	Gene	LNN	MLP	RBF	EC	MDR	Bayes Nets	Naïve Bayes	SVM	Decis. Tb.	DTNB	BFTree	AdaBoost
**SNP** **(1:0)**	**DRD3**	62.9%	59.5%	58.9%	56.6%	60.0%	62.5%	61.6%	64.8%	62.2%	59.5%	61.3%	63.4%
	**HTR2A**	62.4%	62.9%	63.7%	57.5%	64.0%	61.9%	66.6%	65.2%	61.0%	62.3%	62.8%	63.5%
	**Both**	64.5%	64.7%	62.5%	58.7%	64.0%	61.2%	64.8%	64.9%	61.5%	66.2%	62.9%	65.9%
**SNP** **(1:0.5)**	**DRD3**	74.6%	72.9%	71.5%	71.0%	60.5%	71.3%	71.0%	75.4%	73.5%	70.4%	73.7%	71.3%
	**HTR2A**	75.9%	75.5%	73.6%	71.7%	74.2%	62.2%	62.9%	77.4%	73.2%	70.9%	74.5%	71.4%
	**Both**	78.2%	76.8%	74.4%	71.5%	70.7%	62.9%	63.3%	76.8%	73.1%	73.2%	75.0%	71.4%
**SNP** **(1:1)**	**DRD3**	80.5%	79.5%	78.5%	78.2%	69.8%	77.9%	76.2%	81.4%	79.6%	77.1%	79.4%	78.6%
	**HTR2A**	80.7%	81.7%	80.2%	78.5%	71.0%	71.9%	72.3%	83.0%	79.8%	76.8%	81.2%	78.8%
	**Both**	81.4%	82.2%	80.2%	78.6%	71.3%	71.7%	72.0%	82.6%	79.4%	78.5%	81.2%	78.8%
**SNP** **(1:2)**	**DRD3**	87.0%	86.1%	85.8%	85.4%	79.4%	84.8%	83.2%	87.7%	86.6%	80.4%	86.1%	85.2%
	**HTR2A**	88.0%	88.1%	86.3%	85.9%	81.4%	81.3%	81.6%	88.8%	86.5%	76.2%	87.6%	86.1%
	**Both**	87.8%	88.4%	86.5%	85.8%	81.4%	81.3%	81.3%	88.5%	86.7%	79.2%	87.9%	86.1%
**SNP** **(1:3)**	**DRD3**	89.9%	89.5%	88.9%	88.4%	84.8%	89.4%	86.9%	90.6%	89.5%	87.6%	89.5%	88.7%
	**HTR2A**	90.4%	90.7%	89.3%	89.1%	85.9%	85.7%	85.9%	91.4%	89.7%	86.5%	90.3%	89.4%
	**Both**	91.5%	91.3%	89.3%	89.1%	86.1%	85.7%	85.6%	91.2%	89.5%	89.1%	90.9%	89.4%
**SNP** **(1:4)**	**DRD3**	91.9%	91.7%	91.3%	90.9%	87.4%	91.5%	89.2%	92.5%	91.6%	90.3%	91.5%	90.7%
	**HTR2A**	92.6%	92.7%	91.8%	91.2%	88.5%	88.6%	88.6%	93.2%	91.7%	88.5%	92.4%	91.5%
	**Both**	92.6%	93.0%	91.6%	91.2%	89.3%	88.5%	88.5%	93.0%	91.6%	91.1%	92.5%	91.5%
**SNP** **(1:5)**	**DRD3**	93.9%	93.1%	93.0%	92.1%	88.4%	92.9%	90.8%	93.6%	93.1%	91.8%	92.9%	92.2%
	**HTR2A**	93.2%	93.9%	92.9%	92.6%	91.2%	90.5%	90.5%	94.3%	93.1%	90.0%	93.5%	92.9%
	**Both**	93.9%	94.2%	93.1%	92.6%	91.2%	90.4%	90.4%	94.2%	93.1%	92.6%	93.8%	92.9%

*Notes*: LNN = Linear Neural Networks, MLP = Multilayer Perceptron; RBF = Radial Base Functions; EC = Evolutionary Computation; MDR = Multifactor Dimensionality Reduction; Bayes Nets = Bayesian Networks; SVM = Support Machine Vectors; Decis. Tb. = Decision Tables; DTNB = Decision Table Naïve Bayes Hybrid Classifier; BFTree = Best-First decision Tree classifier; AdaBoost = Adaptative Boosting.

**Figure 2:**
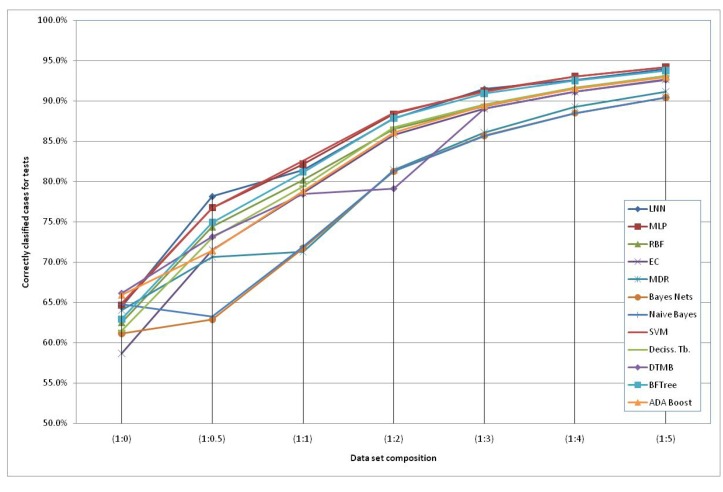
Correctly classified subjects depending on the simulated negative data for both genes; the dataset labels represent the proportion between real subjects (positive and negative = case and control) and simulated negative subjects.

The models generated using the original dataset correctly classify only 66.6% of the schizophrenic subjects when using the HTR2A gene and the Naïve Bayes method. This low accuracy can be due to the reduced number of subjects available and an increased number of “3” values of the SNPs (unknown data). Therefore, we included additional simulated subjects obtained with the HAP-SAMPLE software [15] in the negative group (*non-Schizo*), maintaining the capacity to evaluate positive subjects (cases) for the models. Thus, seven datasets have been created, labeled as SNP (1:*n*), where 1:*n* (*n* = 0, 0.5, 1, 2, 3, 4, 5) is the proportion between the real subjects (positive and negative) and the simulated negative subjects (see details in the Experimental and Theoretical Section). The graphical representation of the evolution of the best classification depending on the additional number of simulated negative subjects is shown in Figure 2. It can observed that the classification percentages do not increase significantly after adding five parts of simulated negative subjects. Among the best models, we propose the following two QGDR models which correspond to simple linear artificial neural networks (LNN).

**Figure 3:**
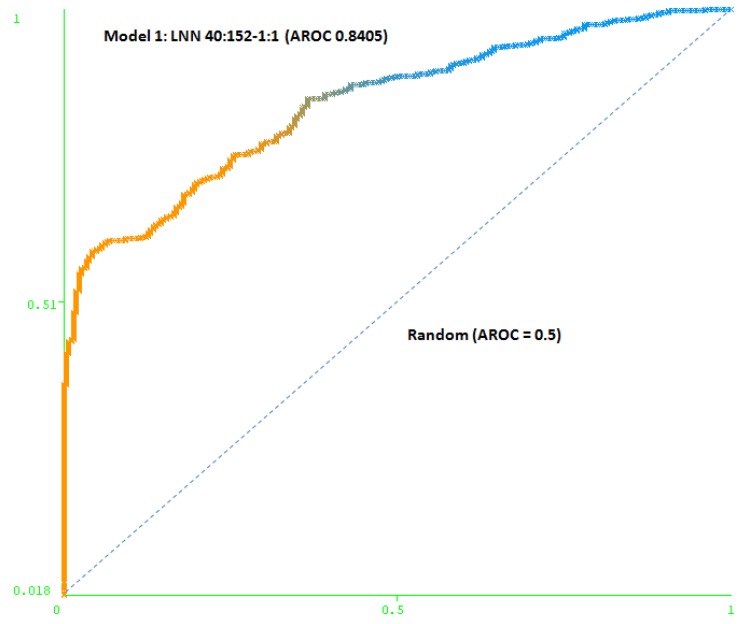
Area under the receiver operating characteristic curve (AUC-ROC) for LNN 40:152-1:1 (Model 1).

The first model (Model 1) includes only a minimum number of simulated negative subjects, SNP (1:0.5): 260 real positive subjects, 354 real negative subjects and 307 simulated negative subjects for schizophrenia, a total of 921 subjects. It is based on 40 SNPs (at DRD3: rs7631540, rs6808291, rs1486012, rs9824856, rs2134655, rs963468, rs3773678, rs167771, rs226082, rs1486009, rs6280, rs7638876, rs9825563, rs1354348; at HTR2A: rs3889066, rs7329640, rs10507544, rs7333412, rs3125, rs6314, rs6308, rs1058576, rs1923884, rs2296972, rs9316233, rs659734, rs1928042, rs2770296, rs582385, rs1928040, rs731779, rs985934, rs9534505, rs6304, rs6305, rs2070036, rs6313, rs1328685, rs731244, rs10507547) and the model used was a LNN with 40 inputs and 152 neurons, which correctly classifies 78.2% of the subjects of the test group. The area under the receiver operating characteristic curve (AUC-ROC) for the cross-validation group (0.8405) shows that the model is not random (see Figure 3). 

**Figure 4:**
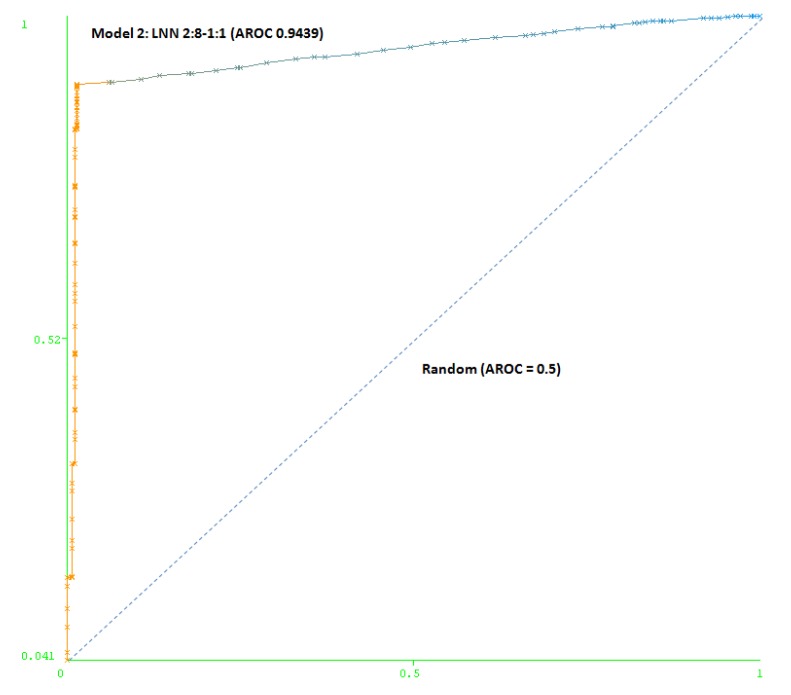
Area under the receiver operating characteristic curve (AUC-ROC) for LNN 2:8-1:1 (Model 2).

The second model (Model 2) includes a maximum number of simulated negative subjects, SNP (1:5): 260 real positive subjects, 354 real negative subjects and 3070 simulated negative subjects for schizophrenia, a total of 3,684 subjects. The model is based only on two SNPs (rs7329640 and rs985934) at HTR2A: a LNN with two inputs and eight neurons, which correctly classifies 93.2% of the subjects of the test group. The AUC-ROC for the cross-validation group (0.9439) demonstrates the goodness of the model (see Figure 4).

Table 1 shows that the classification accuracy percentages are in the range of 56.6–66.6% for SNP(1:0), 60.5–78.2% for SNP(1:0.5), 69.8–83.0% for SNP(1:1), 76.2–88.8% for SNP(1:2), 84.8–91.5% for SNP(1:3), 87.4–93.2% SNP(1:4) and 88.4–94.3% for SNP(1:5). In general, we can observe that the genotype information from the HTR2A gene is classifying more accurately than when considering the SNPs at DRD3 and using the Support Machine Vectors (SVM) technique [45]. There are two exceptions to this performance, with small differences, in the schizophrenia classification for SNP (1:0.5) and for SNP (1:3), where the maximum accuracy percentages correspond to LNN using information from both genes. Despite the fact that an MLP is more complex than an LNN, the first one obtains almost the same classification scores as the LNN. Finally, Evolutionary Computation (EC) [46] obtains better classification scores when the second gene or both genes together are considered, as a higher number of SNPs is taken into account and, thus, there is more information.

## 3. Experimental and Theoretical Section

### 3.1. Subjects and Genotyping

The case-control subjects consisted of 260 unrelated patients (65% males) being treated by the Galician Mental Health Service for schizophrenia and 354 unrelated blood negative donors (45% males) recruited from the Galician Blood Transfusion Centre (staff at the University of Santiago de Compostela and patients attending the University of Santiago de Compostela Hospital Complex). The study protocol was approved by the Bioethics Committee of the University of Santiago de Compostela (for details see [9]). In order to extract genomic DNA from white blood cells of peripheral venous blood from control and case subjects a standard protocol has been used. SNP genotyping was performed using the MassARRAY SNP genotyping system (Sequenom Inc., San Diego, CA, USA) [47]. Re-genotyping of random samples, which represented a total of 600 successfully replicated genotypes, revealed an accuracy rate of >99.9%.

### 3.2. Datasets

Seven datasets have been used containing the following 48 SNPs at the DRD3 and HTR2A genes associated with schizophrenia from the Galician population [9]: rs4682148, rs7631540, rs6808291, rs1486012, rs9824856, rs2134655, rs963468, rs3773678, rs167771, rs226082, rs10934256, rs1486009, rs6280, rs7638876, rs9825563, rs1354348, rs9283560 (17 SNPs at DRD3) and rs3889066, rs7329640, rs10507544, rs7333412, rs3125, rs6314, rs6308, rs1058576, rs6561333, rs1923884, rs2296972, rs9316233, rs659734, rs1928042, rs2770296, rs9316235, rs582385, rs1928040, rs731779, rs985934, rs9534505, rs6304, rs6305, rs2070036, rs2070037, rs6313, rs1328685, rs731244, rs1360020, rs10507546, rs10507547 (31 SNPs at HTR2A). SNPs can take different values: 0 if homozygous (both copies of a given gene have the same allele) for the first allele (one of a number of alternative forms of the same gene occupying a given position on a chromosome), 1 if heterozygous (the patient has two different alleles of a given gene), 2 if homozygous for the second allele or unknown. 

Additional negative subjects have been generated using the simulation tool named HAP-SAMPLE [15]. HAP-SAMPLE is a web application for simulating SNP genotypes for case-control and affected-child trio studies by re-sampling from Phase I/II HapMap SNP data. Providing a list of SNPs to be "genotyped," along with a disease model file that describes causal SNPs and their effect sizes, the application returns two sets of simulated genotypes from case and control subjects. We discarded the case subjects. Thus, a file was created with a different number of control subjects, which were added to case subjects from real clinical data. This data was modified in order to introduce genotyping errors taking into account the error frequencies of the real data.

In addition to the original dataset that contains 260 positive subjects and 354 negative subjects SNP (1:0), we obtained six datasets by including 307, 614, 1,228, 1,842, 2,456 and 3,070 simulated negative subjects. The datasets were named: SNP (1:0.5), SNP (1:1), SNP (1:2), SNP (1:3), SNP (1:4) and SNP (1:5), where the label represents the proportion between the real subjects (positive and negative) and the simulated negative subjects.

### 3.3. QGDR models

The classification models have been obtained with the following methods: Linear Neural Networks, Multilayer Perceptron, Radial Base Functions, Bayesian Networks, Naïve Bayes, Support Machine Vectors, Decision Tables, Decision Table Naïve Bayes Hybrid Classifier, Best-First decision Tree classifier, Adaptative Boosting (all of them from Weka 3.6.2 [48]), Evolutionary Computation and Multifactor Dimensionality Reduction.

**Figure 5:**
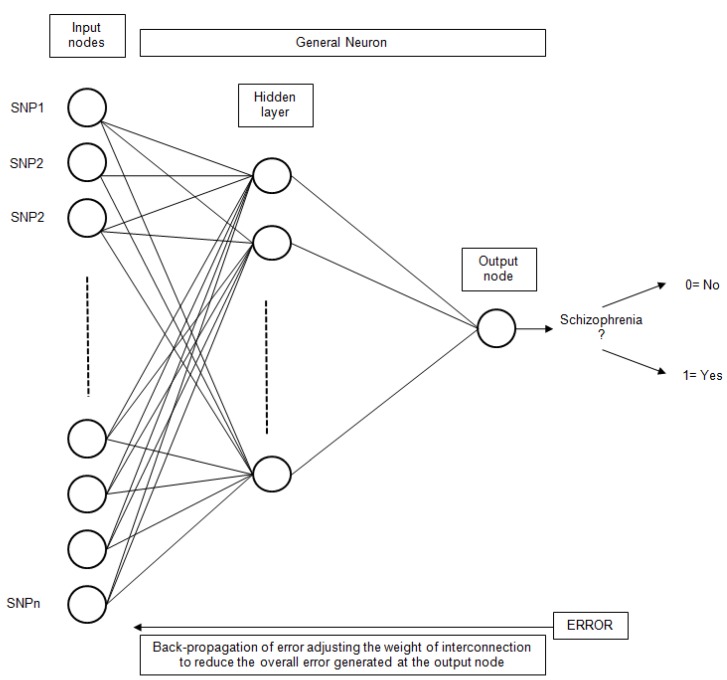
The general structure of an ANN for schizophrenia classification based on SNP inputs.

Artificial Neural Networks (ANN) have been extensively used for classification problems. More specifically, the simple Perceptron [49], also known as Linear Neural Network (LNN), has been utilized. This technique uses a linear network model, with no hidden layers, to perform classification. The Multilayer Perceptron (MLP) [50] has also been utilized. Other types of networks considered were Radial Base Functions (RBF) [51]. In this type of network, the neurons of the hidden layer perform a calculation function instead of the activation function of the MLP. The general scheme for an ANN with only one hidden layer is presented in Figure 5.

As well as the MLP, Support Machine Vectors (SVM) are nonlinear classifiers. SVM induce linear separators or hyperplanes in the space of characteristics. This type of classifier has proved to be very useful when dealing with high dimensionality problems [45]. 

Bayesian methods have also been applied to this problem. These methods are based on Bayes’ theory of probability. Not only they allow performing classification, but they also allow finding relationships among attributes. Several of these methods have been used, such as Naive Bayes [52] (which assumes that the attributes are independent), and Bayesian Networks [53].

The following techniques allow obtaining classification models based on “IF-THEN-ELSE” rules or on hierarchical structures such as trees. More specifically, rule inference models from Decision Tables [54] have been used, building a decision table majority classifier. This type of method evaluates feature subsets using best-first search and uses the nearest-neighbor method to determine the class for each instance that is not covered by the decision table or by the Decision Table Naïve Bayes Hybrid Classifier (DTNB) DTNB [55]. A similar model was used to infer decision trees, following a hybrid approach between the decision trees and the Naïve Bayes classifier, called Best-First decision Tree classifier (BFTree) [56].

Finally, we tried a boosting meta-algorithm. This algorithm consists in combining multiple classification models that complement each other. The Adaptative Boosting (AdaBoost) [57] method builds the models iteratively, weighting the instances differently in each iteration. The new models classify the instances that the previous models do not classify correctly.

Multifactor Dimensionality Reduction (MDR) [58,59] is a data mining approach designed to detect and characterize nonlinear interactions among discrete attributes or variables that influence a binary outcome (for example, case-control status). It is a constructive induction algorithm which reduces the original n-dimensional model to a one-dimensional model, repeating this procedure for each possible n-factor combination and selecting the combination that maximizes the case-control ratio of the high-risk group. This method is considered to be a nonparametric alternative to traditional statistical methods. The MDR software combines attribute selection, attribute construction and classification with cross-validation. This method has mostly been used to detect gene-gene interactions or epistasis in genetic studies of common human diseases [60,61,62] such as schizophrenia [63,64,65], although it can also be applied to other domains.

The technique of Evolutionary Computation (EC) [46] used in this paper is based on genetic algorithms (GAs) [66]. A GA is a search method based on Charles Darwin’s Theory of Evolution [67]. Algorithms based on GAs make a population evolve through random actions similar to those existing in biological evolution (mutations and genetic recombination, as well as selections with a certain criteria called fitness). The fitness is used to decide which individuals are selected, *i.e.,* the more suitable individuals are the higher likelihood they will reproduce. More specifically, the method considered here follows the Iterative Rule Learning (IRL) approach [68,69]. Thus, the result of this method is a set of rules which are used to classify the input data. Like MDR, this method tries to find relationships between attributes or variables and a binary outcome. It has mostly been applied to biomedical data; however, it is still in development.

For each classification, the data has been split into two groups: *Schizo* (positive/case subjects) and *non-Schizo* (negative/control subjects). The SNPs have categorical values of “0” if homozygous for the first allele, “1” if heterozygous, “2” if homozygous for the second allele “3” for unknown genotypes. The 10-fold cross-validation method [70,71,72] has been used to verify the accuracy of the models. The efficiency of the models that evaluate if a patient has schizophrenia is mainly due by the number of correct classifications when using the test set. In addition, these models have been constructed using the SNPs at only one of the two genes or at both of them. Therefore, the classification results have been obtained using 12 machine learning techniques and seven datasets that include different percentages of simulated negative subjects, that is, 252 classification models to be tested.

## 4. Conclusions

This work presents a disease computational study of schizophrenia based on DNA molecule information provided by SNPs and proposes for the first time, to the best knowledge of the authors, two classification models for schizophrenia evaluation. 252 classification models have been obtained using SNPs at two schizophrenia-related genes (each of them or both), twelve machine learning techniques and seven datasets. The best relationships between the DNA molecule sequence and schizophrenia evaluated 78.3–93.8% of the DNA sequence from schizophrenia patients, for datasets with extra simulated negative subjects. In future work, QGDR models will be extended to other types of complex diseases, such as colorectal cancer and cardiovascular diseases, and the best models will be implemented online for free access.
